# Editorial: Responsive biomaterials for controlled release and cancer theranostics

**DOI:** 10.3389/fbioe.2023.1255293

**Published:** 2023-07-19

**Authors:** Nansha Gao, Xiaowei Zeng, Hongzhong Chen, Guoqing Pan, Guojun Chen, Zhongjian Xie

**Affiliations:** ^1^ Shenzhen Children’s Hospital, Clinical Medical College of Southern University of Science and Technology, Shenzhen, Guangdong, China; ^2^ School of Pharmaceuticals Sciences, Sun Yat-sen University, Shenzhen, Guangdong, China; ^3^ Institute for Advanced Materials, School of Materials Science and Engineering, Jiangsu University, Zhenjiang, Jiangsu, China; ^4^ Department of Biomedical Engineering, McGill University, Montreal, QC, Canada

**Keywords:** responsive biomaterials, controlled release, drug delivery, cancer theranostics, cancer nanotechnology, personalized medicine

Nowadays, cancer has become a major public health problem worldwide. Responsive biomaterials undergo changes specifically in response to specific environmental induction, providing potential clinical applications for controlled release, cancer therapeutics diagnostics and personalized medicine. The microenvironment of tumor site is significantly different from that of normal site, such as higher temperature, lower pH value or secretion of certain specific enzymes. Recently, a great quantity of stimuli-responsive biomaterials have been engineered for biomedical applications using different environment stimulus, which could be chemicals such as pH, glucose, enzymes, or physicals such as ultrasound, light, temperature, radiation, or their combinations.

In this Research Topic, we consisted of 10 articles, including six articles, two review articles and two mini reviews, contributed by 67 researchers worldwide. The original research articles involved multiple delivery systems: nanohybrid hydrogels, nanoemulsions, microcapsules and other nanoparticles or nanosheets. These nanosystems were rationally designed and synthesized of novel responsive nanomaterials for controlled release and cancer theranostics or other disease treatments, providing enormous references for their clinical applications.

There are two review articles on this Research Topic. In a review article, Ding et al. provided a systematic summary of photothermal nano hydrogels and discussed their biomedical applications. They noted that the preparation of photothermal nanohydrogels should focus on the photothermal nanomaterials, and summarized the most commonly used photothermal agents including nanomaterials made of metal, carbon based, metal sulfide/oxide, polymer, black phosphorus, MXenes, organic dye and other composite nanomaterials. The authors highlighted the applications of photothermal nanocomposite hydrogel in drug release, photothermal anti-bacterial and wound repair, photothermal inhibition of cancer, bone tissue regeneration, and other aspects such as hydrogel eye piece or electric response hydrogel. In another review article, Qian et al. summarized the advantages and disadvantages of several widely used nanomaterials including mesoporous silica nanoparticles (MSNs), quantum dots (QDs), gold nanoparticles (AuNPs), liposomes, carbon nanotubes (CNTs), and magnetic nanoparticles (MNPs) for the treatment of urinary tract tumor in the areas of controlled drug release, biosensing, tumor imaging and treatment therapy. They pointed out that more clinical studies would be needed to confirm the safety and efficacy of nanoparticles in tumor diagnosis and therapy.

The two mini reviews summarized the graphene-based nanomaterials and chitosan-based hydrogels for stimuli-responsive drug-delivery systems, respectively. Khakpour et al. first introduced the crystal structure and excellent optical, electrical, and thermal properties of graphene-based nanomaterials including graphene oxide (GO) and graphene quantum dots (GQDs). Then, the derivative modification for drug delivery with polymers, biomacromolecules and nanoparticles were discussed. In the end, the authors listed the potential and progress of stimuli-responsive drug delivery from six aspects: pH-sensitive, redox-responsive, ROS-responsive, NIR-responsive, thermo-responsive, and electro-responsive. Garshasbi et al. indicated that injectable chitosan-based hydrogels could offer tremendous potential for drug delivery and tissue engineering due to their better biocompatibility, strong adhesion and hemostatic activity. They dedicated the applications and advantages of injectable chitosan-based hydrogels in cartilage healing, bone tissue engineering, dental pulp stem cells, and anti-cancer drug delivery system. By utilizing the carrier capacity and handling flexibility of *in situ* gelation, they suggested to regulate the dosage of a hydrogel formulation, since the injectable gel must undergo a sol-gel transition near or at the intended insertion site.

In this Research Topic, all of the original studies presented crucial aspects regarding the preparation, modification, and potential clinical translation of responsive biomaterials. pH-sensitive biomaterials take advantage of the lower pH of tumor microenvironment to achieve tumor drug targeting, improving anticancer therapeutic efficacy when combined with photodynamic or photothermal therapy. Combined chemo-photodynamic therapy (chemo-PDT) is always impeded by macrophage clearance and nonspecific distribution in healthy tissues, so extracellular acidity (pH_e_ ∼6.5–6.8) in the tumor matrix is a promising stimulus. Zhang et al. constructed a mixed polymeric micelle (^DA^NP_CT_) that encapsulates chlorin e6 (Ce6) as the photosensitizer and hypoxia-induced prodrug tirapazamine (TPZ), finally achieved controlled photodynamic therapy and hypoxia-activating chemotherapy through pH_e_-induced TAT presentation in the tumor matrix. In an original study, Yin et al. developed a novel drug delivery system using surface modification of polydopamine (PDA) and a folate-targeting ligand, which achieves pH-responsive, long-term circulation *in vivo*, active targeting functions and provides a promising chemotherapy strategy for improving the treatment of oral cancer. The PDA membranes exhibited pH sensitivity and photothermal effect, which facilitated drug release in the acidic tumor microenvironment under laser irradiation, demonstrating remarkable chemotherapeutic-photothermal synergy. In another work, Sun et al. designed a paclitaxel loaded, hyaluronan-conjugated polypyrrole-baced nanoparticle with a cystine dihydrochloride connecting arm for reduction response. It exhibited favorable photothermal effects and enhanced drug release through a combined response to temperature and redox, indicating promising potential for synergistic chemo-photothermal therapy.

One article has been published related to imaging-guided cell tracking. Imaging technologies for tracking cells *in vivo* may provide non-invasive, real-time, quantitative, and multi-dimensional information about the cells. Tang et al. fabricated a partially fluorinated paramagnetic nanoemulsions using perfluoro-tert-butyl benzyl ether, a partially fluorinated agent, for ^19^F MRI-fluorescence imaging (FLI) dual-modal cell tracking, showing improved physicochemical properties while maintaining high ^19^F MRI sensitivity for dual imaging cancer cells tracking.

In addition to improving the anti-tumor therapeutic effect as drug delivery carriers, some progressive response biomaterials also exhibit great potential in the treatment of other diseases. Wang et al. encapsulated magnolol that extracted from magnolia plants to construct poly (DL-lactide-co-glycolide)–poly (ethylene glycol) (PLGA-PEG) nanoparticles, and investigated their preventive and therapeutic effects on OVA-induced chronic asthma in mice. In another article, Xu et al. focused on a lidocaine-embedded polylactic acid-glycolic acid (Lidocaine@PLGA) ultrasonic-responsive microcapsule for relieving sciatica nerve pain. They indicated that the use of ultrasound as a trigger switch could achieve ultrasound-triggered rapid release of lidocaine from the microcapsule.

In conclusion, the current Research Topic reports different types of responsive biomaterials and their broad applications in controlled release, cancer theranostics and other personalized medicine, providing new chances for developing advanced drug delivery systems towards clinical translation.

